# Developing morphological knowledge with online corpora in an ESL vocabulary classroom

**DOI:** 10.3389/fpsyg.2022.927636

**Published:** 2022-07-27

**Authors:** Rui Zhang

**Affiliations:** ^1^Research Center for Linguistics and Applied Linguistics, Xi'an International Studies University, Xi'an, China; ^2^School of English Studies, Xi'an International Studies University, Xi'an, China

**Keywords:** morphological knowledge, online corpora, vocabulary classroom, ESL, community of inquiry, CAR

## Abstract

Morphology is the study of word forms and the ways in which words are varied and related to other words in a language. It has been regarded as an essential discipline that is indispensable in language acquisition. It helps learners to figure out the word structure and meaning, particularly the meaning changing of morphemes, which is pivotal for defining words. The present study focuses on developing morphological knowledge with online corpora which are the useful tools for teaching and learning the changes happened in English. Given this light, this research reports an ESL vocabulary classroom in which the instructor designs vocabulary classroom activities with COCA, BNC, TIME MAGAZINE corpus to enrich students' English vocabulary knowledge, help them master word usage, and foster their corpus literacy. This study is oriented by the framework of Classroom Action Research (CAR). Students' classroom performances were recorded and their self-reflections of learning experiences were collected for thematic analysis. The results indicate that, firstly, students' morphological knowledge has been developed as well as their vocabulary knowledge; secondly, their vocabulary self-regulated learning motivation has been incentivised that they are able to answer their own queries about words; thirdly, students' corpus literacy has been fostered, which facilitates their ongoing vocabulary learning; last but not least, a community of inquiry has been established in which students collaborate to construct vocabulary knowledge. The study has wider implications for constructing the student-centered vocabulary classroom and implementing corpus-based instruction in other second language vocabulary classrooms.

## Introduction

Morphology plays an important role in language learning, which deals with how the word forms. In the context of English vocabulary learning, when learners first learn words, they acquire free morphemes, such as “*seed*”, and they gradually learn about adding a bound morpheme, a suffix “-y”, to form the adjective “*seedy*”. The meaning varies from a plant having “*gone to seed*” to “*shabby*”. This morphological process is involved in the learners' lexical acquisition in which learners examine how the suffixes extend the meaning of roots as well as generate different parts of speech. As Nagy et al. (1993) point out, students' morphological knowledge of prefixes, roots, and suffixes is fundamental for understanding new words. A lot of research suggests that morphological knowledge facilitates the process of comprehending, storing, and retrieving words (Bowers and Kirby, 2010; Kucan, 2012; Templeton, 2012, 2017; Akbulut, 2017; Goodwin et al., 2020). Therefore, it can be assumed that the more insight the learners have into morphological knowledge, the deeper understandings they can get in their vocabulary learning. Corpora are the useful teaching tools which provide authentic contexts of lexis used in diachronic order. Learners can explore morphological meaning changing and the use of words through concordance lines, whereby they can not only learn the word usage but also explore the historical meaning changing of morphemes. In the morphological process, learners learn vocabulary *via* deducing the word structure and inferring the word meaning with morphological knowledge, and this is endorsed by a couple of researchers that morphological knowledge is a multi-dimension (i.e., semantic, syntactic, phonological, and orthographic information) for learners to explore the nature of words presented as the morphological units (Wolf, 2007; Templeton, 2017; Goodwin et al., 2020).

Many researchers focus on corpus-assisted vocabulary teaching and learning mainly from language production and teaching plan (Frankenberg-Garcia, 2014; Ko and Goranson, 2014; Soruç and Tekin, 2017; Khan, 2019; Rana and Amin, 2020; Ma and Mei, 2021), but few of them concentrate on developing morphological knowledge *via* implementing corpora, which is crucial for vocabulary learning. The present study draws attention to applying online corpora to English vocabulary teaching and learning with the purpose of addressing the following research questions:

1. Is students' morphological knowledge developed with online corpora? What morphological knowledge do they acquire?
2. How do online corpora influence students' vocabulary learning?

The results from students' presentations and self-learning reflections evidently show that students' morphological knowledge has been expanded through exploring the historical meaning changing of morphemes with online corpora. In the vocabulary classroom, students enrich their vocabulary with morphemes and morphemic meanings. Their self-regulated learning motivation has been incentivised that they are encouraged to use online corpora to search words and figure out meanings independently, and they are able to answer their own queries about words *via* the corpora they select. In addition, students' corpus literacy has been fostered, which facilitates their ongoing vocabulary learning. Based on the findings, the present study demonstrates that the corpus-based approach has wider implications for constructing the student-centered vocabulary classroom in ESL and other second language vocabulary classrooms.

## Morphology and corpora

Morphology is known as the study of word formation, focusing on morphemes which are the smallest meaningful units of a language (Anderson, 1982, 1992, 2015; Haspelmath and Sims, 2010; Lieber, 2016). It involves inflection and derivation of morphemes that are closely related to the process of learners' lexical acquisition. As some researchers argue, information about the meaning, pronunciation, and part of speech of a word is derived from its morphological knowledge (Nagy et al., 1993; Stoffelsma et al., 2020; Goodwin et al., 2022). In particular, morphological knowledge is a fundamental dimension for discovering what is in a word, through which learners deduce the word forms and access the morphemes, i.e., prefixes, roots, and suffixes, and these are essential for learners to develop the generic knowledge of vocabulary (Schreuder and Baayen, 1995). When inferring the meaning of a word, morphological meaning changing is crucial since it influences the word formation in ways of derivational regularity and historical borrowing (Anderson, 2015). For example, the suffix “-nik” in English lexis is firstly derived from Russian which means “little”. The word sputnik was found frequently used in the 1950s in the TIME Corpus since the Soviet Union launched the first artificial satellite into space, and it was called Sputnik meaning “little moon” in Russian. Its derivational adjectives such as udarnik (highly-productive shock-brigade worker) and voyentechnik (technician) mean the professionals; beatnik refers to the declined generation of rebellion youth and is related to some kind of fanatic in the 1960s; refusenik indicates a Soviet citizen in the late 1960s when social revolution burst in America, and it was in this period, “-nik” has come to be associated with a sense of communist values. The 1970s onwards show the survival of the main coinages (e.g., kibuzznik) and the arrival of some new ones, but the generative power of the suffix is fading. It can be noticed that languages change as time goes by. During the changing process, new morphemic units come into existence and the established ones may fade away along with the tides of fashion (Baayen, 2009). Such historical meaning changing of morphemes is preferable to be tracked and explored through corpora.

Corpora, which have generally been viewed as a large sample of naturally occurring language, written or spoken, stored in the computer and that can be searched using different kinds of software (Biber et al., 1998; Sinclair, 2005; McEnery and Hardie, 2012; Weisser, 2016; Anderson and Corbett, 2017). Corpora provide a huge number of authentic texts of words and their usage in a real context searched in its context of use, but it does not directly present the meaning of a word which has to be deduced from the concordance lines generated (Q'Keeffe et al., 2007).

The abundance of authentic texts is closely related to the language culture and its linguistic patterns, providing real materials for language learners and this is pivotal for lexical acquisition (Reppen, 2011). In a corpus, a word and its context are displayed in diachronic order, providing social and historical information about each lexical item——when it first appears, what genre it is applied to, and where it is found. This information, “metadata” (Anderson and Corbett, 2017), is helpful for learners to explore morphology from the aspects of derivational regularities and historical changes, and it is in line with what Stefanowitsch (2020) maintains, that is, ‘corpus morphology is mostly concerned with the distribution of affixes, and retrieving all occurrences of an affix plausibly starts with the retrieval of all strings potentially containing this affix' (p. 309).

The corpus-based approach is salient for doing diachronic research, offering interesting results when it is used to study language changes. On the bases of corpora, it is palpable that through concordance lines, the context of society, culture, history, and community has been richly glossed, and it is also apparent to identify types containing the affix in concern and review how the words are structured. Like the aforementioned “-nik”, it is transparent to figure out its meaning changing and its combinability through the concordance lines, and it is straightforward to explore the language changes in a natural speech community. By going top down the concordance lines, learners can analyse how the senses shift according to how the words and expressions are used in particular grammatical constructions. Therefore, it can be assumed that using corpora in vocabulary teaching can enable students not only to recognize words but also to analyse their introvert structure systematically and historically with the purpose of facilitating vocabulary teaching and learning.

## Corpus-assisted vocabulary teaching and learning

A number of researchers conducted empirical studies on corpus-assisted vocabulary teaching and learning in the second language learning context (Frankenberg-Garcia, 2014; Ko and Goranson, 2014; Soruç and Tekin, 2017; Khan, 2019; Rana and Amin, 2020). Frankenberg-Garcia (2014) tested the usefulness of separate corpus examples for English comprehension and production among Portuguese secondary school students. The findings show that a single corpus example and multiple corpus examples can help language comprehension. The study also obtains detailed separate encoding and decoding examples to provide evidence in support of corpus-assisted language learning, particularly when learners need different types of examples. However, the question remains to be answered, that is, how to provide learners with easy access to multiple corpus examples mainly on the patterns of language that they need to look up for language production. Soruç and Tekin's (2017) did a qualitative study investigating 26 learners' perceptions of corpus-assisted vocabulary learning activities based on COCA and BNC corpora in an EFL classroom in Turkey. The study collected data *via* reflection papers, semi-structured interviews, and a personal evaluation scale. The results indicated that learners find corpus-assisted vocabulary learning activities interesting, innovative, autonomous, and practical. As Johns (1994) argues, data-driven learning brings learners to authentic language use and corpus rightly holds the feature which helps students develop their learning strategies outside of the classroom with technology. Ko and Goranson (2014), and Rana and Amin (2020) did pre-test and post-test to examine the effectiveness of using corpora in English vocabulary teaching. Their findings suggest that students who are taught by the corpus-based approach performed much better. From their comments, they applaud that corpus provides multiple forms of information for learning new words than guessing the meaning of words from a single context. Moreover, students' productive knowledge increased significantly, and their vocabulary learning motivation has been strengthened.

Other scholars put forward practical and effective corpus-assisted vocabulary teaching plans. Khan (2019) designed a corpus-assisted vocabulary teaching plan to teach four-word lexical bundles to ESL students at a community college in the USA. The plan suggests that the application of BYU-iWeb corpus for the selection and instruction of four-word lexical bundles helps students attain accuracy and proficiency in their writing expressions. The design enables students to work together by using lexical bundles to produce texts in their academic writing. Ma and Mei (2021) reviewed corpus tools for vocabulary teaching and learning, illustrating how corpora benefit vocabulary teaching and learning and how we choose appropriate corpora. The researchers introduce Corpus-Based Language Pedagogy (CBLP), suggesting designing corpus-based lessons with four design principles—“detecting lexical errors”, “observing and analyzing the language”, “summarizing the language use pattern”, and “practicing using the language”. They also pointed out some issues for teachers to consider in their teaching design—providing students guidance when requiring them to work with corpora, balancing the use of corpora and non-corpora resources, and creating opportunities for students to use the words in context.

As for teaching word formation, a lot of researchers focus on using corpus data to measure morphological productivity and derivation (Baayen and Lieber, 1991; Nagy et al., 1993; Plag and Baayen, 2008; Baayen, 2009; Schröder and Mühleisen, 2010; Fernández-Domínguez, 2013). A few researchers draw attention to implementing corpora in teaching morphology, a pivotal knowledge in vocabulary learning. Wu (2014) discussed the necessity of using corpora in teaching morphology in ESP courses. She argues that corpus data can improve learners' morphological productivity which in turn assists learners in memorizing ESP vocabulary. Petrovitz and Pierson (2018) analyzed the Chinese English Corpus (CEC) encompassing Chinese international students' entrance-examination essays. The findings suggest that students have mastered standard deviational morphology, but simply for correcting language forms and it rarely benefits creativity and effective communication.

Up to this point, there has been little research on the empirical studies of using corpora as a tool for students to develop morphological knowledge, particularly the historical meaning changing of morphemes, which is essential for learning words. The present study demonstrates that online corpora are useful tools for exploring the historical meaning changing of morphemes and this enhances vocabulary teaching and learning.

## Research design

This study is situated in the framework of Class Action Research (CAR), a methodology widely used in the pedagogical field to improve classroom teaching and learning (Burns, 2010; Mettetal, 2012; Mills, 2018). It incorporates informal research practices such as presentations, teaching observation, and learning reflections. Rather than focusing on the statistical or theoretical significance of findings, triangulation of data analysis is performed for validity. By conducting the CAR, students' learning achievements and their learning reflections help teacher-researchers discover the effectiveness of the teaching technique, and this will influence the teacher's teaching refinement which in turn affects students' learning. The characteristic of the CAR takes the form of a dynamic cycle of teaching that brings teaching into a more concrete and efficient orientation than would be the case with purely abstract theorizing. The CAR involved in the present study takes the elements mentioned above into the instructor's consideration: (1) Identifying a research focus. (2) Data Collection. (3) Taking Actions. (4). Reflection.

## Identifying a research focus

In the previous English classroom, the teacher found that, firstly, students have issues in identifying the meaning of words ended up with infrequently used suffixes, such as “*-ista*”, “*-nik*”, “*-vik*”, etc.; secondly, students get panicked when some senses of words are not listed in the dictionary. For example, “*seedy*”, the “sexually unsavoury” element of its meaning has not found its way into the online *Oxford English Dictionary* where the closest senses are “shabby” or “ill as a result of excessive drink”. Moreover, students are not activated in vocabulary learning. In other words, they are reluctant to develop the knowledge of lexis which is important for lexical acquisition. As Sinclair (1991), one of the pioneers of modern corpus linguistics, noted that ‘the language looks rather different when you look at a lot of it at once.' (p. 100). Based on these preliminary observations, the present research then focuses on using online corpora to facilitate students' morphological learning and vocabulary enhancement, and to invoke their English vocabulary independent learning motivation.

## Data collection

Data were collected from 31 students, third-year undergraduates, majoring in English. Among them, 12 are male and 19 are female. The class is for helping them effectively acquire English vocabulary. The author is the teacher-researcher teaching the class. The students attend the course voluntarily. They all signed the consent form. They know that they are engaged in the study and they have the right to end their participation in the research at any time without penalty. The Vocabulary Self-collection Strategy (VSS) chart is used to list the word selection process by students. VSS can promote students' vocabulary learning interest through students' participation in a learning community, in which students share knowledge with words and strengthen self-efficacy in vocabulary learning. According to Haggard (1982), by implementing VSS, students are encouraged to find words and determine the meaning by themselves, and in the next-day presentation, students need to tell where the words are found, what the context-derived meanings are, and why they think the class should know the words. Other students and the teacher discuss about information to reach an agreement on the meanings. After the discussion, a word list is accomplished, and students will have a vocabulary journal at the end of class for review. Students' learning reflections are used to record their learning experience about using online corpora in their vocabulary learning activities.

## Taking classroom action

The vocabulary classroom action is taken according to the following procedure adapted from Yanto and Nugraha (2018):

1. Scaffolding

The instructor primarily trains students to use online corpora, such as the Corpus of Contemporary American English (COCA), the TIME Magazine Corpus, and British National Corpus (BNC). After the corpus training, the instructor introduces VSS to students and guides them to make VSS charts with the purpose of initiating students to know about the vocabulary strategy and familiarizing them with the steps: (a) select words from the reading materials, (b) reasons for the selected words, (c) students' definitions of the words, (d) corpus' information of the words and usage. The VSS chart is presented in Table 1.

**Table 1:** VSS chart sample.

**No**	**Word**	**Source of the word**	**Reasons for selection**	**Students' definition**	**Corpus' information**	**Word usage**
1						
2						
3

2. Group discussion

Students are divided into small groups consisting of 2–3 students each. In the group discussion, students discuss the meanings of words, the reasons for choosing these words, and the importance of words for comprehending the reading content. They show the teacher reading content and the words that they find interesting or hard to understand, and then complete the VSS chart with their group members. Each group offers 2–3 words and the whole class has around 35 words to learn in total.

3. In-class presentation

Each group selects a presenter to do the class presentation with PPT slides, reporting the information about their selected words. Other students and the teacher together have a discussion and record the selected words and the contextual meanings in the VSS class chart. The whole class then has an integrated word list for vocabulary learning. Table 2 is one of the groups' VSS charts.

**Table 2:** Student group's VSS chart.

**No**s	**Word**	**Source of the word**	**Reasons for selection**	**Students' definition**	**Corpus's information**	**Word usage**
1	Hemophilia	News	Medical terminology, illness	Blood disorder	The royal disease; effects of the bleeding disorder, including frequent hemorrhages and debilitating pain	Noun and no plural forms; collocated with words like transmit, dread, suffer, etc.
2	Pedophilia	News	Same suffix but the negative meaning	Abnormal hobby	the upsurge of cracking down on internet pedophile pornography crimes	Frequently used with the words like crime, church, study, etc.
3	RUSSOPHILIA	News	Political tendency	People who are friendly toward Russia or fond of Russia and Russian things	Someone who is sympathetic to the political system and customs of the former Soviet Union	Collocated with Trump, flavor of, communist, etc.

**Figure 1:**
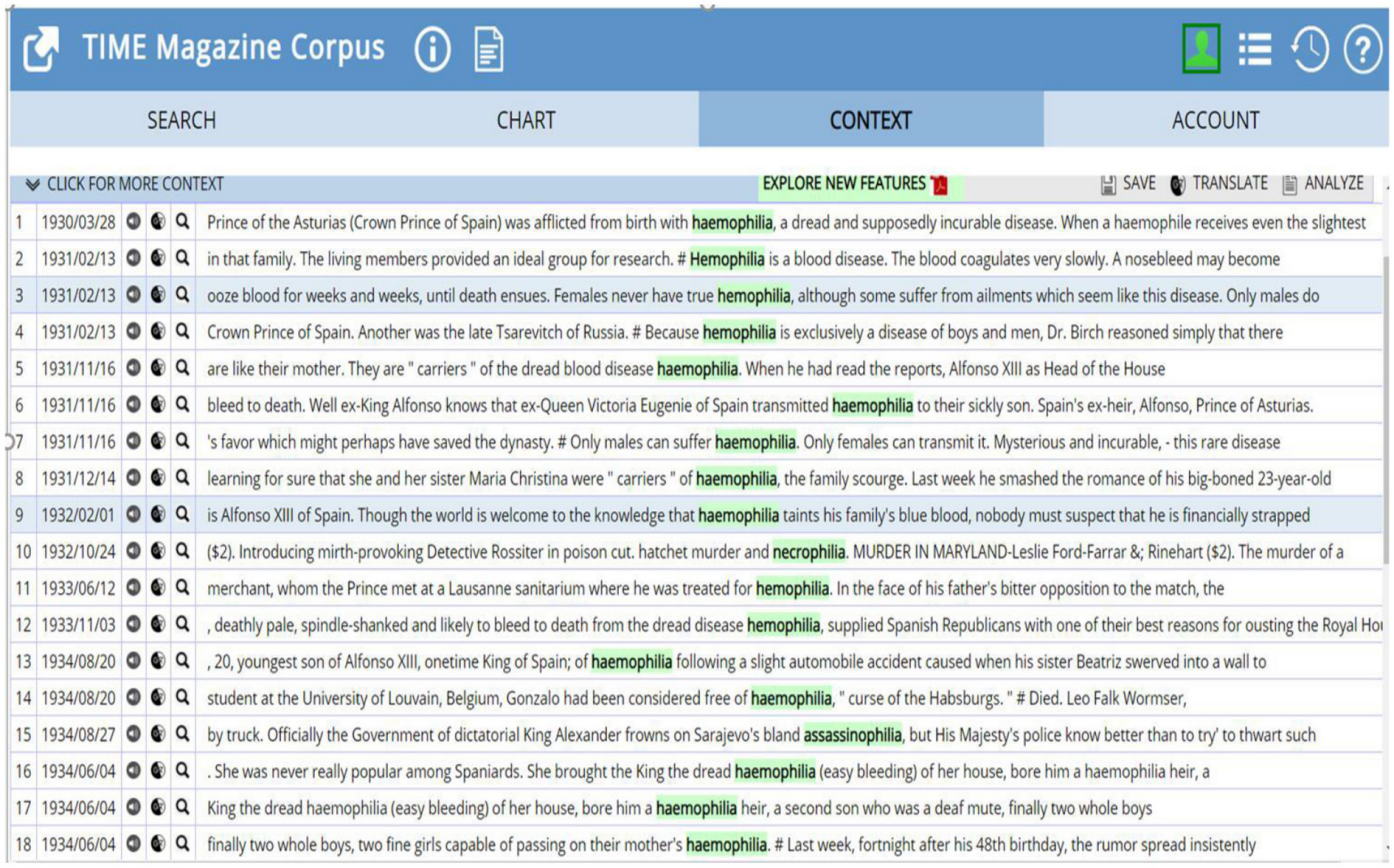
Concordance lines of h(a)emophilia.

The group presenter shared her group attainment in class with the PPT slides about their learned words. They came across the word “*Socialphobia*” in the web news. When looking for the meaning of “*-phobia*”, the students find another suffix “*-philia*” which has a similar form but opposite meaning to “*-phobia*”. They looked up “*-philia*” in the *Online Etymology Dictionary*, noticing that it is from Greek, meaning “friendship, fondness, tendency toward” and in recent use “abnormal attraction to”. The students are curious to know the words end up with “*-philia*”, its historical information, and its contextual usage. Accordingly, they used the online corpora, COCA and TIME Magazine Corpus, to explore the information they are interested in. The findings are mainly discussed in Figure 1.

From the Figure 1 in the TIME Magazine Corpus, “*hemophilia*” is the only word that appeared most frequently and there are 34 relevant contexts with the suffix “*-philia*” in the 1930s. It was first introduced to the world as “The Royal Disease” during the reign of Queen Victoria of England. She was a carrier of the hemophilia gene. However, it was her son, Leopold, who suffered the symptom of the bleeding disorder, including frequent hemorrhages and debilitating pain. Unfortunately, Leopold passed the carrier gene to his children who eventually married into the royal families of Russia, Spain, and Germany, extending the condition throughout the European Royal bloodlines. This is the key information to illustrate why “*hemophilia*” connects with royalty in the concordance lines, and it collocates with “*suffer*”, “*dread*”, “*transmit*”, etc.

**Figure 2:**
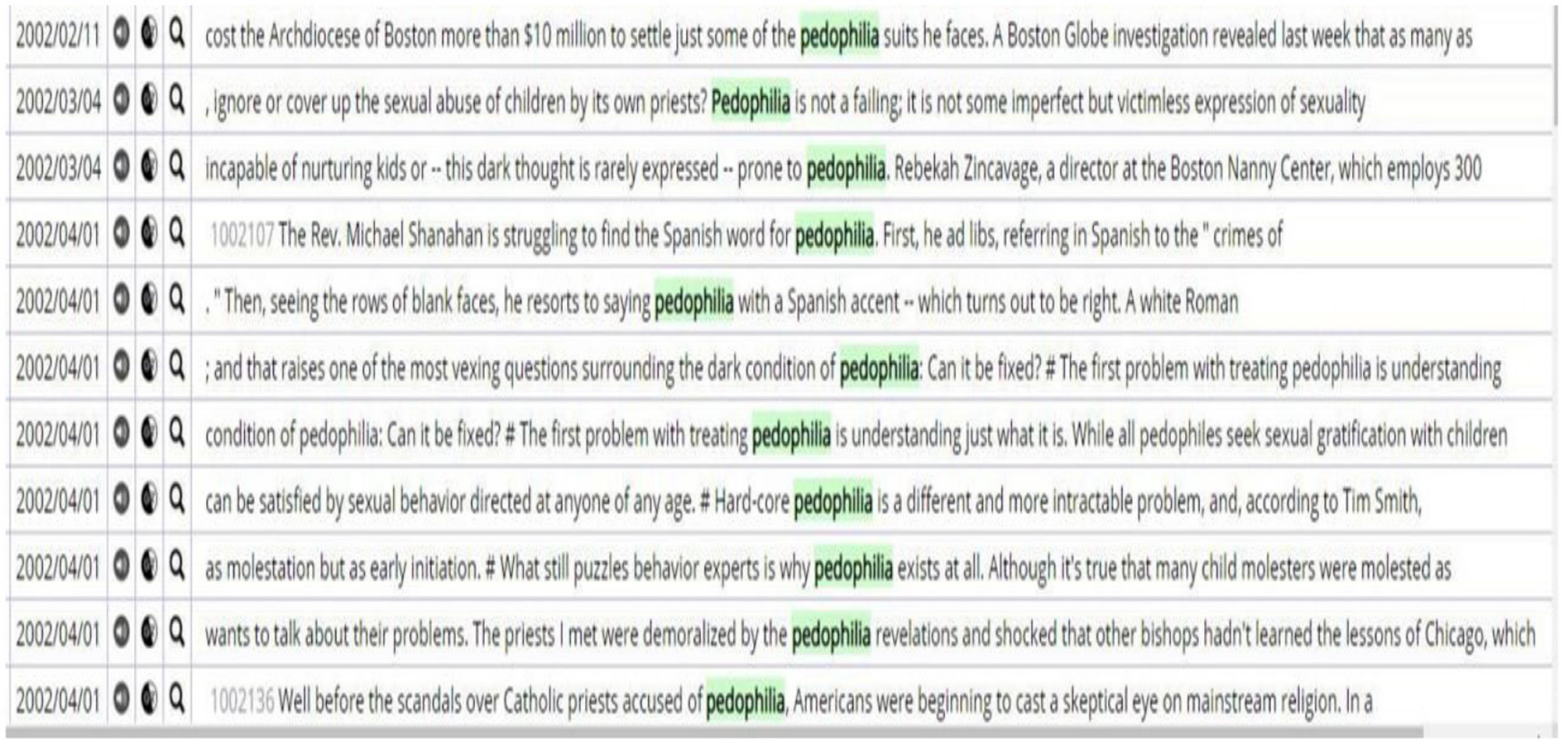
Concordance lines of pedophilia.

The word “*pedophilia*” came into being at the end of the nineteenth century and widely appeared in the 2000s as the Figure 2 shows.

Particularly after the 1990s, with the upsurge of cracking down on internet pedophile pornography crimes, “*pedophilia*” has gradually become a hot topic in public. In 2003, the United States successfully filmed a documentary that is related to the most sensational case of child molestation in American history. This documentary undoubtedly makes everyone better understand this topic. Moreover, the development of the internet in the last 10 years of the twentieth century allows the proliferation of child pornography. In 1977, 1990, and 1994, the United States promulgated laws to punish pedophilia. Accordingly, it can be found from the concordance lines that “*pedophilia*” frequently appears with “*crime*”, “*sexuality*”, “*accuse of* ”, etc. In 1982 and 1990, the Federal Supreme Court found evidence related to pedophilia when handling some cases. Therefore, “*pedophilia”* became a public topic widely and was frequently used after the 1980s. In addition, it is surprisingly found that “*pedophilia*” also collocates with “*treatment*”, and this indicates “*pedophilia*” is also treated as a disease.

**Figure 3:**
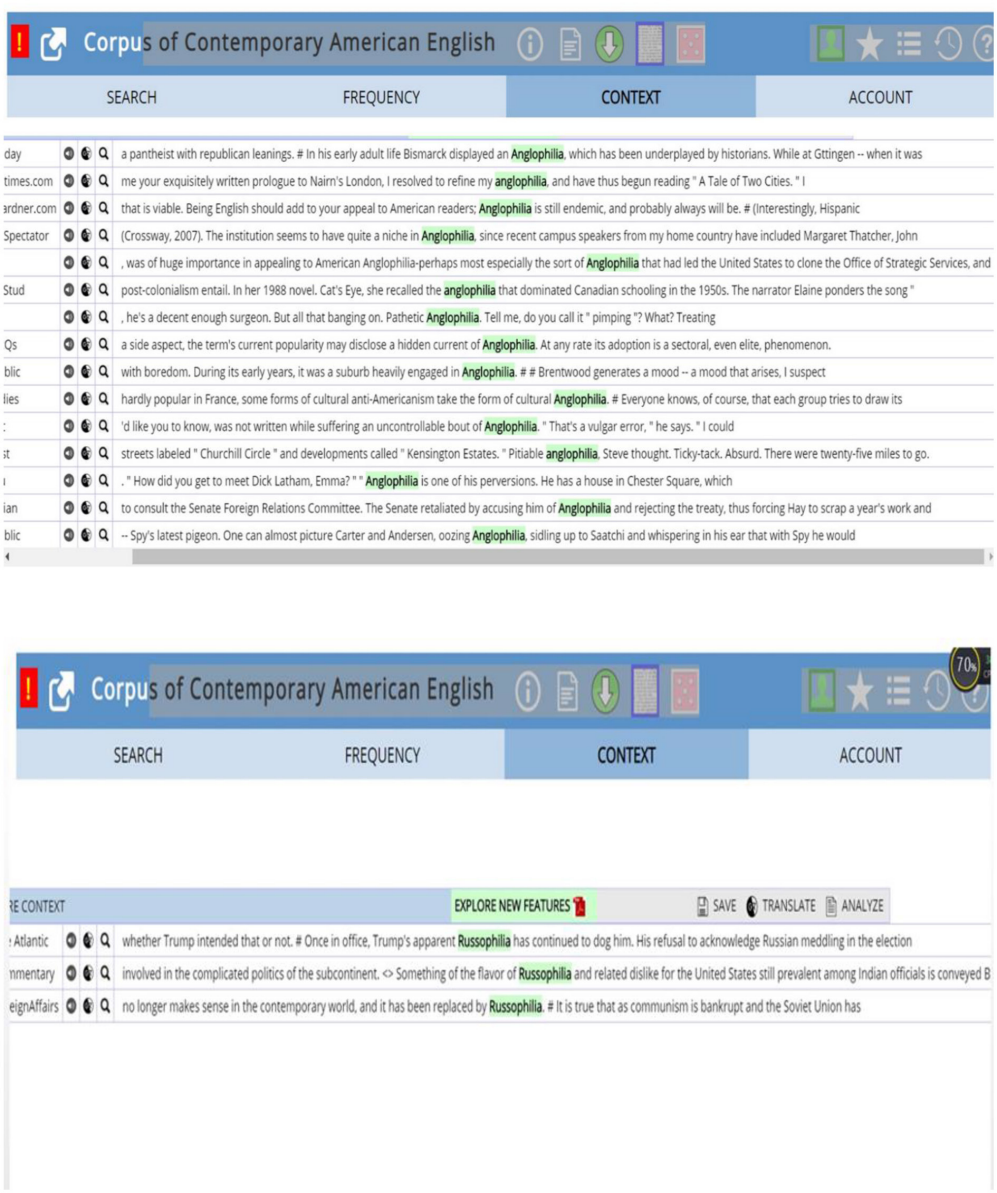
Concordance lines of Anglophilia and Russophilia.

From the 1960s to the 1990s, some words are structured in the form of “country's name + -philia”, for example, “*Anglophilia*”, “*Russophilia*”, “*Europhilia*”, etc., see the searching results from COCA (Figure 3).

Why these kinds of words suddenly emerge in the 1960s? This is also the question asked by other students. After introducing the background information of that period, it is discovered that the social situation rightly accounts for the phenomenon. In the late 1950s, the main strength of the capitalist camp differentiated into a tripartite confrontation as America, Japan, and Europe, while the Socialist camp began to split. Therefore, the international political paradigm took up a multi-polarization mode. Under this circumstance, most countries all over the world had their own aligns, and the words, *Anglophilia, Russophilia*, and *Europhilia*, appear timely. These words are used adjacently with “*flavor*”, “*support*”, “*communist*”, “*oozing*”, etc. from the concordance lines.

After the in-class discussion, all the students have got a vocabulary list and a vocabulary journal at the end of the semester for them to review whenever and wherever they want. Most importantly, in the process of exploring the word information *via* online corpora, students learn the words along with developing the morphological knowledge and this is completely different from the traditional vocabulary learning in way of content, mode, and cognition. Students' learning experiences and achievements in morphology and vocabulary were recorded in their reflective journals.

## Data analysis

Thematic analysis (Braun and Clarke, 2006) was used to identify motifs occurring in the content of students' learning reflections to demonstrate students' learning achievements and to account for the usefulness of assisting students' vocabulary learning and morphological knowledge. Data were first assigned *in-vivo* codes. These *in-vivo* codes were then grouped under patterns, through which the themes were identified by comparing shared meanings and characteristics. The coding was done by the author from 35 texts and there were 27 distinct codes generated, through which 4 themes were identified. Results of each coding were checked by a research assistant to test the reliability of the coding system and the validity of the coding process, in which the codes were appropriately grouped under the thematic categories.

## Results and discussion

### Students' learning reflections

Thirty-one students completed their learning reflections which were coded under the themes of: (a) morphological awareness, (b) vocabulary learning motivation, (c) corpus literacy, (d) community of inquiry. The classification of students' learning reflections is shown in Table 3.

**Table 3:** Classification of students' learning reflections.

**Categories (student number)**	**Examples**	**Proportion**
Morphological awareness (SN = 30)	Morphemes are important historical knowledge of morphemes is fantastic morphemes influence the meaning and the structure of words suffixes are complex but important for the meanings of words knowing morphemes is helpful for memorizing the meanings of words word knowledge has been expanded through learning morphemes	96.77
Vocabulary learning motivation (SN = 28)	Enjoy learning English vocabulary motivated to learn new words competent in memorizing words and their meanings like using online corpora to search words learning activities are interesting will learn more words independently	90.32
Corpus literacy (SN = 29)	Access to authentic language usage gain more information than in the dictionary apply it to discourse analysis convenient to analyse data a basic resource for language research challenging and rewarding expect to know more about corpora	93.55
Community of inquiry (SN = 27)	Share findings and understandings with group members collaborate to search words with corpora encourage to learn more words discussions help me clarify my thinking together to resolve word problems	87.10

#### Morphological awareness

From Table 2, it is noticeable that 96.77 percent of students stated that in the vocabulary learning process, students became aware of the importance of morphological knowledge, including morphemes and morphological meaning changing when defining the meanings of words and analyzing the word formation. Excerpts are shown below:

*Student 1. Before, I did not pay attention to morphemes nor pay much attention to the word structure. I just consulted the word meaning from the dictionary and wrote it down. … Now, I know morphemes are important in vocabulary learning*.

*Student 2. Morphemes, particularly suffixes are complex but now I find them important for figuring out the meanings of words. … It's easy to use corpora to clarify the meanings of affixes. I will use corpora to look for more words that have the same affixes. It is fun*.

*Students 3. Historical knowledge of morphemes is fantastic. I now understand that it influences the meanings and the structures of words. … I am now curious about searching for the words that end up with the same suffixes with online corpora and exploring their historical meaning changing*.

*Students 4. There are a lot of morphemes I don't know. I will look for more in my vocabulary learning and search for their meanings with online corpora. … Knowing morphemes is helpful for memorizing words and meanings. … My word knowledge has expanded through learning morphemes*.

From the students' reflections, it is evident that students are aware of the importance of morphology in their vocabulary learning, particularly the meaning changing of morphemes. A lot of students stated that historical knowledge of morphemes influences the word structure and meaning. They reflect that it is fun to learn morphemes. As students 2, 3, and 4 mentioned, using online corpora to search words ending up with the same suffixes and exploring the meaning changing of them *de facto* help with defining the words. Moreover, students acknowledge that their word knowledge has been expanded through morphological knowledge. As shown in the in-class presentation, students develop their word knowledge *via* searching in the online corpora to enlarge their vocabulary stock. In a word, students' morphological awareness has been formed.

#### Vocabulary learning motivation

Regarding motivation of vocabulary learning, 90.32 percent of students' learning motivation has been activated and their independent learning has been invoked in way of responding to their own inquiries about vocabulary. Excerpts are displayed as follows:

*Student 5. … The learning activity is interesting. Making the VSS chart is very helpful for learning words. In the beginning, it's not easy, because finding words and finalizing the word list are time-consuming and skill required. However, it is worth trying. I now enjoy learning English vocabulary*.

*Student 6. In the vocabulary classroom, I feel that I become competent in memorizing words and their meanings. I now like using corpora to search words and their usage. … My performance in the learning process is good and I did well in the class presentation. I believe that I can do better in the vocabulary learning activities*.

*Student 7. It is fun to learn English words with online corpora. I can find words and understand the meanings by myself. … Now, when I come up with new words, I can settle them by myself. I can learn more words independently*.

The above reflections reveal that students' English learning motivation has been incentivised. Although some students feel pressure and have difficulties when they use the VSS chart and online corpora for the first time, they eventually become skillful and satisfied with their attainments in their vocabulary learning. It is apparent that students' independent learning has been reinforced that they use corpora to learn words, memorize them, and figure out their meanings and usage by themselves. It can be safely concluded that corpus-based approach pedagogically integrated technology and classroom vocabulary teaching. In addition, students' self-efficacy was fulfilled. They are pleased with their learning performances and believe that they can do better in the vocabulary learning activities. This is in accordance with the research conducted by Zimmerman et al. (1996) that self-regulated learning can enhance self-efficacy. It also demonstrates that self-efficacy is an important element in vocabulary acquisition, influencing independent learning and metacognition which are useful for learners (Mizumoto, 2012).

#### Corpus literacy

About 93.55 percent of students show that they accept and fancy online corpora. They are able to search for word information from the authentic language text in the online corpora, and they would like to know and use more corpora to do research. Below are excerpts:

*Student 8. It's a little bit challenging to use online corpora at first, but it is rewarding. I can find more information about words in the online corpora than in the dictionary. … Online corpora provide various kinds of information, such as collocation, colligation, and data-driven grammar. It enriches my vocabulary knowledge*.

*Student 9. Online corpora open a new world to my English study, allowing me to look at the language usage from quite a novel perspective. The language text that I see from the online corpora is authentic and it is used in an actual context. … I am intrigued by the corpora and I want to do discourse analysis with them*.

*Student 10. Online corpora show me the material of the language, spoken or written, by the native speakers. It provides a platform where I can gain a better understanding of word usage. … I now understand MI score can be used to look at the co-occurrences of words and can be compared across corpora. … I want to know more about corpora to do my research, conversational discourse. It's a basic resource for language research and convenient to analyse data*.

The students' reflections suggest that they are capable to find word usage with online corpora, such as collocation, colligation, data-driven grammar. They also learned that MI (mutual information) score is used to measure co-occurrences of words and it can be compared across corpora of different sizes. Remarkably, online corpora enable students to access the authentic language used in a real situation and this is the intriguing part that attracts students. Remarkably, some students expect to know more corpora to do research. As students 9 and 10 mentioned, they would like to do discourse analysis and corpora are useful tools for analyzing data. All in all, students' corpus literacy has been well-cultivated, they desire to use corpora not only to enrich their English vocabulary but also to do language research, and this, I argue, is the great effect and achievement of implementing corpora in the vocabulary learning classroom to promote students in their continuous learning.

#### Community of inquiry

Results showed that around 87.10 percent of students reckon that in-class presentations and group discussions are beneficial for sharing findings and understandings, collaborating for word search, exploring information, and resolving learning problems. Excerpts are presented as follows:

*Student 11. In the group discussion, we discuss the word list and share our findings and understandings about word information.... I talk to my group members when I don't understand the findings. Discussions help me clarify my thinking when I feel confused*.

*Student 12. I like group work. When I have problems in using online corpora, my group members will help me. We together solve problems*.

*Student 13. In the class presentation, I share my search results with other students and have a discussion with them and the teacher. They ask me some questions and the teacher make some comments about my presentation. … I have learned a lot*.

The above excerpts notably illustrate that in the process of vocabulary learning, a community of inquiry (Garrison and Vaughan, 2008) has been established, in which students share their findings and understandings of words, settle disputes, gain knowledge about vocabulary, and help each other when coming up with corpus problems. Students, in this vocabulary learning classroom, not only acquire vocabulary knowledge but also cultivate the ability of resolution which profoundly benefits them in their professional study.

As aforementioned, students' learning reflections show that online corpora help with establishing students' morphological awareness. The morphological awareness discussed in this research has shifted the focus from consciousness and ability to the morphemic structure of words aiming to expand and deepen students' word knowledge. It is aligned with Bowers et al. (2010) and (Lyster et al.'s, 2013) argument that morphological knowledge is indispensable to consolidating and refining students' vocabulary learning. Moreover, by using corpora, students' intrinsic learning motivation is incentivised. They are active to look for words and learn by themselves, and this is better than traditional vocabulary teaching—asking students to look up words in the dictionary and memorize them. As Ryan and Deci (2000) maintain, intrinsic motivation is the inherent tendency to meet the challenges by which learners' learning competence is developed. Therefore, corpus-assisted language learning is an effective approach for teachers to activate students' morphological learning motivation which in turn facilitates their vocabulary acquisition.

Students' learning reflections also indicate that their corpus literacy has been cultivated. They understand what the corpora are, know how to analyse corpora data and how to extract findings from that data. In general, they are motivated to use corpora to learn words and some of them are curious to do language research through corpora. It can be argued that corpora are not only tools for teachers to use in the vocabulary classroom but also a way to arouse students' academic curiosity, through which students are initiated to the linguistic research domain and this manifests the effect of implementing corpora in the language learning classroom. However, students may still need teachers' instructions about corpus utilization, particularly in corpus selection and data sorting. Therefore, teachers should pay more attention to students' corpus-used issues in their learning process to ensure that corpus-based data-driven learning can largely benefit students' language acquisition, and this is also in accordance with Ma et al.'s (2021) point of view that teachers should provide detailed guidance when implementing corpora as resources to address students language learning needs, such as showing the case about selecting corpus, exemplifying data sorting, categorizing results, etc., and in the teaching process, teachers are expected to create sufficient opportunities for students to deal with their target language issues through corpora.

During the collaborative learning process, the community of inquiry has been set up in which students communicate and share their understandings with each other, and develop solutions to problems that can be applied in practice. This collaborative learning environment offers students a learning atmosphere that enables them to negotiate their thinking with shared understandings to construct knowledge. Pedagogically, it is the most rewarding outcome that develops solutions to coursework problems in a community of inquiry (Arbaugh et al., 2008; Garrison, 2017). Overall, based on the results of students' learning reflections, it is apparent that the conduct of corpus-based data-driven learning considerably promotes students' morphological knowledge exploitation, in the process of which students collaborate with each other, make decisions about intended learning words, search for relative information, share and negotiate with peers, present their attainments in class, and this is a cyclical follow supporting their continuous learning.

## Conclusion

This study attests the effectiveness of applying the corpus-based approach in an ESL vocabulary classroom. The praxis is oriented by the framework of CAR. The research findings verify that students' morphological knowledge has been developed and expanded with online corpora. During the learning process, students' morphological awareness has been built. They use morphological knowledge to analyse word structure and infer word meaning, which in turn assist them to memorize words and to practice using them. In this sense, online corpora have enriched students' word inventory and knowledge. Although some students have problems when they first try the online corpora, they soon master them and perform well in the vocabulary activities. They adore using online corpora to search words and plan to use corpora to do discourse analysis, even though doing discourse analysis involves corpus building in another linguistic area—“corpus-based discourse analysis”. Notwithstanding, it reveals that students are aware of applying their acquired knowledge in the vocabulary classroom to their English professional study. Furthermore, the community of inquiry has been established in general. Students collaborate to accomplish learning activities, in the process of which they share their understandings, negotiate personal thinking with each other, and construct knowledge. In this way, students' professional knowledge skills are promoted, self-regulated learning motivation is activated, self-efficacy is incentivised, and their professional study expectation is encouraged (Zhang, 2020). Additionally, the study reexamined the practical usefulness of the VSS chart and this could be kept implemented in other second language vocabulary classrooms.

Corpus-assisted vocabulary learning is a new and profound pedagogical approach that modifies EFL/ESL vocabulary classroom teaching. It provides ample authentic language resources for teachers and students to explore morphological knowledge and word formation. However, there are still some limitations when using corpora in the vocabulary classroom. Firstly, some online corpora (e.g., COCA, BYU-BNC, TIME Magazine Corpus, etc.) need an academic license for searching for a number of words. Otherwise, students in classes will be blocked after the total number of queries from the class exceeds 250 searches in a 24-h period, and this might be an issue for universities that have no funds for this expense. Secondly, students might be confused for the first time when exploring the morphological knowledge of vocabulary as there are millions of concordance lines. Teachers can select and tailor the concordance lines for their better understanding.

## Data availability statement

The original contributions presented in the study are included in the article/supplementary material, further inquiries can be directed to the corresponding author/s.

## Ethics statement

The studies involving human participants were reviewed and approved by the Academic Committee of School of English Studies at Xi'an International Studies University. The patients/participants provided their written informed consent to participate in this study.

## Author contributions

The author confirms being the sole contributor of this work and has approved it for publication.

## Funding

This research was supported by the Chinese Educational Ministry Industry—University Cooperative Project under Grant number [202002079007]; Shanghai International Studies University, Institute for Language Materials Development Teaching Materials Research Project under Grant number [2022TX0001]; Xi'an International Studies University Teaching Reform Fund under Grant number [XWK21YB14].

## Conflict of interest

The author declares that the research was conducted in the absence of any commercial or financial relationships that could be construed as a potential conflict of interest.

## Publisher's note

All claims expressed in this article are solely those of the authors and do not necessarily represent those of their affiliated organizations, or those of the publisher, the editors and the reviewers. Any product that may be evaluated in this article, or claim that may be made by its manufacturer, is not guaranteed or endorsed by the publisher.
