# Corrigendum: Developing morphological knowledge with online corpora in an ESL vocabulary classroom

**DOI:** 10.3389/fpsyg.2022.1057275

**Published:** 2022-10-18

**Authors:** Rui Zhang

**Affiliations:** ^1^Research Center for Linguistics and Applied Linguistics, Xi'an International Studies University, Xi'an, China; ^2^School of English Studies, Xi'an International Studies University, Xi'an, China

**Keywords:** morphological knowledge, online corpora, vocabulary classroom, ESL, community of inquiry, CAR

In the published article, there was an error in the **Funding** statement as published. The funding protect was stated as “Shanghai Foreign Language Education Press Teaching Material Research Project” but should be “Shanghai International Studies University, Institute for Language Materials Development Teaching Materials Research Project.” The correct statement appears below.

## Funding

This research was supported by the Chinese Educational Ministry Industry—University Cooperative Project under Grant number [202002079007]; Shanghai International Studies University, Institute for Language Materials Development Teaching Materials Research Project under Grant number [2022TX0001]; Xi'an International Studies University Teaching Reform Fund under Grant number [XWK21YB14].

The authors apologize for this error and state that this does not change the scientific conclusions of the article in any way. The original article has been updated.

## Publisher's note

All claims expressed in this article are solely those of the authors and do not necessarily represent those of their affiliated organizations, or those of the publisher, the editors and the reviewers. Any product that may be evaluated in this article, or claim that may be made by its manufacturer, is not guaranteed or endorsed by the publisher.

